# REL-1017 (esmethadone; d-methadone) does not cause reinforcing effect, physical dependence and withdrawal signs in Sprague Dawley rats

**DOI:** 10.1038/s41598-022-15055-3

**Published:** 2022-07-06

**Authors:** Jack Henningfield, David Gauvin, Francesco Bifari, Reginald Fant, Megan Shram, August Buchhalter, Judy Ashworth, Ryan Lanier, Marco Pappagallo, Charles Inturrisi, Franco Folli, Sergio Traversa, Paolo L. Manfredi

**Affiliations:** 1 Pinney Associates, Bethesda, MA USA; 2 Charles River Laboratories, Wilmington, MA USA; 3 Department of Medical Biotechnology and Translational Medicine, University of Milan, Milan, Italy; 4 Altreos Research Partners, Toronto, ON Canada; 5 Relmada Therapeutics, Inc., Coral Gables, FL USA; 6 Department of Health Sciences, University of Milan, Milan, Italy

**Keywords:** Neuroscience, Neurotrophic factors

## Abstract

REL-1017 (esmethadone, d-methadone) is the opioid-inactive d-isomer of racemic d,l-methadone. REL-1017 may exert antidepressant effects via uncompetitive *N*-methyl-d-aspartate receptor (NMDAR) channel block. As REL-1017 is expected to exert central nervous system activity, full characterization of its abuse potential is warranted. We evaluated lack of reinforcing effect, physical dependence, and withdrawal of REL-1017 in Sprague Dawley rats. (1) *Self-administration Study* Rats were trained to self-administer oxycodone intravenously (IV) and then were subjected to 3-day substitution tests where saline, oxycodone, and REL-1017 were self-delivered IV by a fixed number of lever presses; (2) *Drug Discontinuation Study* Rats were treated for 30 days by oral gavage with vehicle, REL-1017, ketamine or morphine and evaluated for withdrawal with functional observational batteries (FOBs). In the self-administration study, rats treated with saline, vehicle, and all REL-1017 doses showed the typical “extinction burst” pattern of response, characterized by an initial rapid increase of lever-pressing followed by a rapid decrease over 3 days. Rats treated with oxycodone maintained stable self-injection, as expected for reinforcing stimuli. In the withdrawal study, REL-1017 did not engender either morphine or ketamine withdrawal signs over 9 days following abrupt discontinuation of drug exposure. REL-1017 showed no evidence of abuse potential and did not engender withdrawal symptomatology.

## Introduction

REL-1017 (esmethadone; d-methadone) is the dextro-isomer of racemic d,l-methadone and is a novel promising candidate for the rapid treatment of major depressive disorder (MDD). Preclinical studies showed efficacy in all tested murine models of depressive-like behaviour^1,2^. In a Phase 2 trial, REL-1017 demonstrated safety, and was well tolerated, and free of opioid or psychotomimetic effects^3^ in line with Phase 1 results^4^. In patients with MDD and inadequate response to one to three courses of antidepressant treatment, REL-1017 25 and 50 mg orally once daily showed rapid and robust antidepressant effects that were sustained for 1 week after discontinuation of treatment^3^. The rapid antidepressant activity of REL-1017 is thought to be due to N-methyl-d-aspartate receptor (NMDAR) uncompetitive channel block with downstream BDNF and mToR-dependent mechanisms^2,5^. Additionally, REL-1017 has been shown to increase BDNF plasma levels in healthy volunteers in a Phase 1 trial^6^.

The opioid agonist effects of methadone isomers are stereo-selective^7^, while NMDAR antagonistic actions are not^5^. Only levomethadone is active at the opioid receptor, while both isomers, levomethadone and esmethadone are NMDAR blockers^5,7^. The Food and Drug Administration (FDA) recommends abuse potential assessment for all CNS active drugs. Therefore, despite the reported lack of opioid agonist effects of esmethadone in animal models^8–11^ and in humans^12–14^, we tested REL-1017 for its ability to cause reinforcing effects and physical dependence and withdrawal signs in Sprague Dawley rats. Experimental studies of self-administration and physical dependence in rats and non-human primates are based on established experimental models and can help predict the potential for abuse of central nervous system (CNS) active drugs in humans^15–17^.

The rat self-administration assay is considered the gold standard procedure to assess relative abuse potential in vivo^18^. The maintenance of drug-taking behaviors in normal Sprague Dawley rats represents one of the major predictive assays used during the risk assessments conducted by both international^19–24^ and national drug control policy makers, and it is a critical factor in schedule control actions initiated by the FDA and Drug Enforcement Administration (DEA) during the new drug application process. The present study was designed following the FDA’s 2017 Guidance: Assessment of the Abuse Potential of Drugs^24^. In the self-administration study, the procedure involved Sprague Dawley rats trained to stably self-administer oxycodone. The subsequent 3-days substitution session tested a broad range of doses of oxycodone and REL-1017, including REL-1017 doses approximately 3 times higher than the human therapeutic equivalent doses. Similarly, in the physical dependence and withdrawal study, adequate doses of morphine, ketamine, and REL-1017 along with vehicle control were administered for 30 consecutive days, and discontinuation signs were measured for 9 days following study drug discontinuation.

## Methods

This work was conducted in accordance with the U.S. Department of Health and Human Services, Food and Drug Administration, United States Code of Federal Regulations, Title 21, Part 58: Good Laboratory Practice for Nonclinical Laboratory Studies and as accepted by Regulatory Authorities throughout the European Union (OECD Principles of Good Laboratory Practice), Japan (MHLW), and other countries that are signatories to the OECD Mutual Acceptance of Data Agreement. This study was authorized by the Study Director of Charles River Laboratories, Inc. 54943 North Main Street Mattawan, MI 49071, USA. This work has been audited by Quality Assurance of Charles River Laboratories, Inc. 54943 North Main Street Mattawan, MI 49071, USA, in accordance with the applicable Good Laboratory Practice regulations. The study protocol was reviewed and approved by the Institutional Animal Care and Use Committee Charles River Laboratories, Inc. 54943 North Main Street Mattawan, MI 49071, USA.

### Animals

Seven- to nine-week-old male Sprague Dawley rats weighing between 215 and 260 g from Charles River Laboratories were individually housed in solid bottom cages with non-aromatic bedding. The housing was equipped with an automatic watering valve with ad libitum access to water and food (#5002, PMI Nutrition International, Inc.). Housing was maintained in a temperature (68 °F to 79 °F), humidity (30% to 70%), and light-controlled (12/12-h dark/light) environment. As environmental enrichment may be an experimental confounder in studies specifically designed to establish and maintain stable self-delivered drugs of abuse, we used durable (e.g., nylon rod) or non-durable (e.g., nestlets, cocoons) enrichment throughout the study. All procedures were conducted in accordance with the Institutional Animal Care and Use Committee and the Eighth Edition of the Guide for Care and Use of Laboratory Animals (National Research Council 2017) and the ARRIVE Guidelines 2.0 for reporting animal research.

### Test drugs

Esmethadone hydrochloride (Batch-Lot Number 2003000065), cocaine hydrochloride (Batch-Lot Number 1907000055), and oxycodone hydrochloride (Batch-Lot Number 2007000244) were provided by SpecGx LLC. Morphine sulfate (Batch-Lot Number 1IG0879 and 1JA0513) was provided by Spectrum Quality Products. Ketamine hydrochloride (Batch-Lot Number 1IB1041) was provided by Spectrum Chemical MFG Corp. All drugs were stored at controlled room temperature and protected from light. Dosing formulations were prepared to meet dose level requirements.

### Data analysis

Data are shown as the mean ± standard deviation (SD). All data were tested and passed the normality test (Fig. S1). In study 1 (Self-Administration Study), simple linear regressions (slope) were calculated and fitted to the total number of injections, total drug intake, and response rate in the test/substitution session. In study 2 (Drug Discontinuation Study), for each measure, the two variables (treatment and time) of each rat group were compared using two-way analysis of variance (ANOVA). To compare aggregate measures of performance across treatments, we also calculated the area under the curve (AUC) for each treatment measure during the 9 days of drug discontinuation. Statistical differences between groups were calculated with one-way ANOVA corrected by Tukey’s test. p < 0.05 was considered statistically significant.

## Study 1: Self-Administration Study

**Figure 1:**
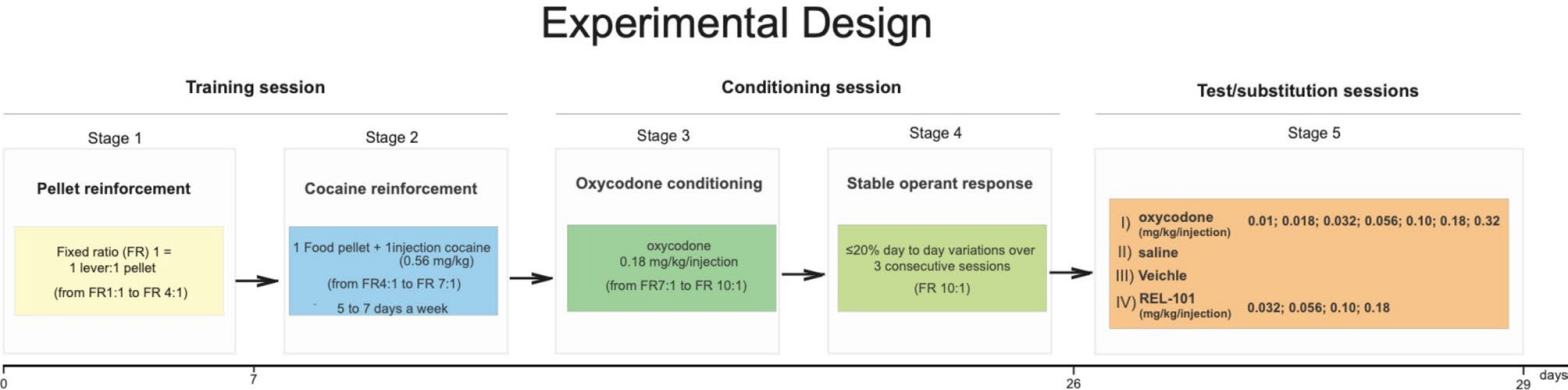
Self-administration study design (Study 1). The drawing illustrates Study 1 experimental protocol. During the first 7 days, rats were trained to respond by foot pressing on a lever, located within an operant response chamber, to receive a single food pellet reinforcement (45 mg). In this initial training session, rats were trained to respond under a fixed ratio (FR) 1:1 (1 lever press = 1 pellet delivery) schedule of reinforcement. When rats consistently responded to food rewards, the response requirements were increased until rats consistently responded under an FR4 (4 lever presses = 1 pellet delivery) schedule of reinforcement (Stage 1). Then, rats were trained to respond on the lever to self-administer cocaine (0.56 mg/kg/injection) paired with the delivery of a single 45 mg food pellet/injection for 1 training session; thereafter, food was discontinued, and the rats continued with self-administration of cocaine only. The progressive establishment of cocaine as a reinforcer was obtained with a final FR7 (7 lever presses = 1 cocaine delivery) (Stage 2). At this point, the conditioning session started, the cocaine was replaced, and the rats were given access to 0.18 mg/kg/injection of oxycodone. During the oxycodone only training sessions, the response requirements were increased until rats achieved a FR10 (10 lever presses = 1 oxycodone injection) (Stage 3). Stable operant responding (Stage 4) was defined as responding under an FR10 schedule for oxycodone injections with ≤ 20% day to day variations over 3 consecutive sessions. Stable responding rats started the Stage 5 operant testing sessions with saline or oxycodone, (“test sessions”), and with the test article or its vehicle (“substitution sessions”). Once a day for three consecutive sessions, lever pressing resulted in self-administration of saline or oxycodone at 0.01, 0.018, 0.032, 0.056, 0.10, 0.18, and 0.32 mg/kg/injection (Test Sessions), and REL 1017 at 0 (vehicle), 0.032, 0.056, 0.10, and 0.18 mg/kg/injection (Substitution Sessions).

The study design is depicted in Fig. 1. Rats were ordered from the supplier with surgically-implanted chronic indwelling jugular vein catheters per their own standard operating procedures^25^. All rats were allowed a recovery period before their shipping to the test facility. During the recovery period, catheters were flushed daily with 50 units of heparin (Sigma-Aldrich, St. Louis, MO) dissolved in saline. Upon arrival in the test facility, all rats were vested in protective jackets designed for catheter-based dosing. Jackets were inspected and changed as needed throughout the study. Catheter exteriorization sites were inspected daily, and catheters were regularly flushed with normal sterile saline. Catheters were also flushed immediately prior to and after self-administration infusion sessions.

Self-administration training and testing procedure were carried out as previously described^18,26^. The rat self-administration procedure that has been adopted by the industry and FDA is described as a single lever operant lever press response under a fixed-ratio 10 (FR10) schedule of drug deliveries with session lengths of at least 1 h duration.

Here we briefly describe the study protocol:

- *Stage 1 (pellet reinforcement)* Rats were trained to respond by foot pressing on a lever, located within an operant response chamber, to receive a single food pellet reinforcement (45 mg). Initially, rats were trained to respond under a fixed ratio (FR) 1 (1 lever press = 1 pellet delivery) schedule of reinforcement. Training sessions were a maximum of 30 min in duration in stage 1, with a maximum of 50 food rewards attainable. When rats consistently responded for the maximal attainable amount of food rewards, the response requirements were increased until rats consistently responded under a FR2 (2 lever presses = 1 pellet delivery) to FR4 (4 lever presses = 1 pellet delivery) schedule of reinforcement.
- *Stage 2 (cocaine reinforcement)* Subsequently, rats were allowed to respond by lever pressing to obtain self-injections of cocaine (0.56 mg/kg/injection) paired with the delivery of a single 45 mg food pellet (1 pellet and 1 injection) for 1 training session. Thereafter, food was discontinued, and rats pressed the lever to obtain cocaine injections. The food/drug pairing training session and all cocaine infusion training sessions had a maximum duration of 30 min in stage 2, with a maximum number of allowed rewards of 10 (reward = 1food pellet/1 cocaine injection).
- *Stage 3 (oxycodone conditioning)* Following the progressive establishment of cocaine as a reinforcer with a final FR7 (7 lever presses = 1 cocaine dose delivery), oxycodone-only training (0.18 mg/kg/injection) sessions started. The self-administration requirements for oxycodone were progressively increased up to a FR10 (10 lever presses = 1 oxycodone injection). Sessions were terminated after a maximum of 60 min, or a maximum of 10 self-injections, whichever occurred first. A 10-s time-out was imposed between the end of a self-injection and the opportunity to respond for another self-injection.
- *Stage 4 (stable operant response)* Rats demonstrated day-to-day stability in self-administration of 0.18 mg/kg/injection of oxycodone. Stable responding was defined as a response with an FR10 (10 lever presses = 1 oxycodone self-injection) with ≤ 20% day-to-day variation over 3 consecutive days.
- *Stage 5 (test substitution session)* Once each rat achieved stable responding, substitution sessions in 6 rats per dose were initiated with vehicle (Sterile Water for Injection [USP]) different doses of oxycodone (0.01, 0.018, 0.032, 0.056, 0.10, 0.18, and 0.32 mg/kg/injection), and increasing doses of REL-1017 (0.032, 0.056, 0.1, and 0.18 mg/kg/injection). Operant sessions were terminated after 1 h without restrictions on the total number of drug self-deliveries earned by the rat during the 3 consecutively scheduled test daily sessions. The total dose of REL-1017 (0.18 mg/kg/injection) delivered during the test session correspond to 7.3 ± 4.3 (mean ± SD) mg/kg/bodyweight. The amount of REL-1017 administered in the rat is approximately 3 times higher than the human equivalent dose that is administered to achieve a therapeutic dosage of 0.42 mg/kg.

Drugs were administered 5 to 7 days per week during both self-administration training and testing sessions.

In summary, in rats with self-administration of oxycodone, we instituted test sessions in which oxycodone was either maintained or substituted with different drugs. In three daily consecutive sessions, rats self-administered the following: (I) oxycodone: 0.01, 0.018, 0.032, 0.056, and 0.18 mg/kg/injection; (II) saline; (III) vehicle; or (IV) REL-1017: 0.032, 0.056, 0.1, and 0.18 mg/kg/injection (Table 1). Animal assessment included behavioral self-administration measures (total number of injections, total drug intake, and response rate), in addition to mortality, body weight, and clinical observations. Clinical observations included evaluation of the skin, fur, eyes, ears, nose, oral cavity, thorax, abdomen, external genitalia, limbs and feet, respiratory and circulatory effects, autonomic effects such as salivation, nervous system effects including tremors, convulsions, reactivity to handling, and unusual behavior. Clinical observations were performed daily during the reinforcement study. Clinical observations in laboratory animals are standardized by both US regulatory guidelines of the FDA and Environmental Protection Agency as well as Internationally through the adoption of the International Council for Harmonisation (ICH) Guidelines. Clinical observations in these studies were based on a standardized listing in general Investigational New Drug (IND)-enabling protocols conducted under Good Laboratory Practice.

**Table 1:** Study design.

Treatment	Dose (mg/kg injection)	Concentration (mg/kg)	Dose volume (μL/injection)	No. of male animals
Saline control	0 (saline)	0	32 to 48	29
Positive control	0.01 (oxycodone)	0.07	37 to 42	6
Positive control	0.018 (oxycodone)	0.126	37 to 42	6
Positive control	0.032 (oxycodone)	0.224	37 to 41	6
Positive control	0.056 (oxycodone)	0.392	38 to 44	6
Positive control	0.10 (oxycodone)	0.70	39 to 43	6
Positive control	0.18 (oxycodone)	1.26	33 to 47	29
Positive control	0.32 (oxycodone)	2.24	40 to 45	6
Test article vehicle	0		38 to 45	6
Test article	0.032 (REL-1017)	0.224	39 to 46	6
Test article	0.056 (REL-1017)	0.392	40 to 46	6
Test article	0.1 (REL-1017)	0.70	41 to 47	6
Test article	0.18 (REL-1017)	1.26	40 to 51	6

The characteristics that defined a test condition as a “positive reinforcer” was operationally defined as previously described^27,28^ and incorporated in the protocol for this study*:* “If responding, as measured by the total number of injections, declines over the course of the three-day substitution period or there are “vehicle-like” or “saline-like” response topographies during the course of testing (e.g. a downward staircase pattern), then the test article will be considered lacking reinforcement properties”.

Animals that successfully demonstrated stable operant responding for oxycodone were utilized on study as indicated in Table 1.

## Study 2: Drug Discontinuation Study

**Figure 2:**
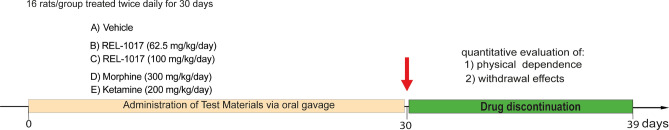
Chronic drug exposure and withdrawal study design (Study 2). All drugs (morphine, ketamine, REL-1017, and vehicle control) were administered daily by oral gavage for 30 consecutive days. In the afternoon of Day 30, all rats received vehicle (sham) doses as their last delivered dose on study to test for withdrawal over 9 consecutive days. A total of 96 rats were tested, 16 rats for each treatment group. During the 9 days of discontinuation to assess physical dependence and withdrawal effects, we assessed neurobehavioral screening battery consisting of a set of functional observational batteries (FOBs), which included measures of activity/arousal, autonomic and physiological domains, and measures of neuromuscular activity (see “Methods” section).

Physical dependence is an adaptive process in response to chronic exposure to certain drugs. The present study was designed and conducted in accordance with the 2017 FDA Guidance on the Assessment of Abuse Potential of Drugs^24^ to assess the potential of REL-1017 to engender physical dependence and/or withdrawal symptoms/signs (Fig. 2, Drug Discontinuation Study Design). Morphine and ketamine were evaluated as positive control drugs. To assess the potential of REL-1017 to produce opiate- or phencyclidine (PCP)-type of physical dependence and withdrawal symptoms/signs, we tested the presence of a discontinuation syndrome in rats following abrupt discontinuation of treatment after 30 consecutive days of drug administration. Each rat on study was assigned to one of five independent groups of 16 rats per group treated by oral gavage twice daily (Table 2).

**Table 2:** Treatment assignment for assessing physical dependence and withdrawal symptoms/signs with REL-1017 and comparators.

Group	Treatment	Dose level (mg/kg/day)	Dosage (mg/kg/dose)	Number of male rats
1	Saline	0	0	16
2	REL-1017	62.5	62.5 qd	16
3	REL-1017	100	100 qd	16
4	Morphine	40 to 300	20 to 150 bid	16
5	Ketamine	200	100 bid	16

The study design is depicted in Fig. 2. The different test drugs (morphine, ketamine, REL-1017, and vehicle) were administered twice daily by oral gavage for 30 consecutive days. Dosing sessions were initiated at 06:30 and 17:00 (± 30 min) so as not to interrupt the 12:12 light/dark cycle. To obtain the final dose of 300 mg/kg/day of morphine, rats were treated twice a day, at an initial oral dose of 20 mg/kg/administration (40 mg/kg/day). Morphine doses were increased up to a maximum of 150 mg/kg/gavage twice daily (300 mg/kg/day). Ketamine was administered at a fixed dose of 100 mg/kg/gavage twice daily (200 mg/kg/day). Vehicle and REL-1017 at 0, 62.5, and 100 mg/kg/day in a 5 mL/kg volume were administered once daily at 06:30 h (± 30 min); the second dose at 17:00, was a vehicle sham dose. The two doses of REL-1017 (62.5 and 100 mg/kg/day) greatly exceed the proposed human maximum daily dose—a 25 mg oral REL-1017 daily dose (approximately 0.35 mg/kg for a 70 kg human) presently undergoing Phase 3 clinical trials in humans. The 100 mg/kg dose of ketamine twice a day was selected because 100 mg/kg dose is the anesthetic dose of ketamine in rats. The 150 mg/kg dose of morphine twice a day was selected because its discontinuation is reliably associated with withdrawal^29^. In the afternoon of day 30, all rats received vehicle (sham) doses as their last dose in order to test for signs of a discontinuation syndrome (drug withdrawal) during 9 consecutive days.

Over the course of the study, rats were evaluated for withdrawal with a series of functional observational batteries (FOBs)^30–33^. Evaluations occurred prior to the initiation of dosing (predose/baseline) and on days 1, 15, 30, and for 9 days following cessation of treatment, during the expected period of “withdrawal” of the study. The FOBs included the following measures: (i) activity/arousal; (ii) sensory-motor activities; (iii) autonomic and physiological domains; and (iv) neuromuscular activity. Automated and validated LocoMotor Activity Monitor systems were used over the first 12 h of “lights-out” following abrupt cessation of 30 days of daily treatments in each of the 96 rats that completed the dosing phase of the study^34,35^. Rats were placed into the infrared photobeam monitoring boxes at approximately 1700 to 1800 h on day 30, just prior to the scheduled “lights-out” period in the vivarium. The rats were then monitored overnight until approximately 07:00 h on day 1 of withdrawal.

### Activity/arousal domain

To assess the level of unprovoked activity/alertness, each animal’s arousal levels were quantified during observations of the unperturbed subject in the open field, with a range of severity scores from coma to hyper-alertness (very depressed = 0 [stupor, coma], depressed = 1 [sluggish, some head or body movement], slightly depressed = 2 [somewhat sluggish, some exploratory movements with periods of immobility], normal = 3 [alert, exploratory movements], slightly elevated = 4 [slight excitement, tense, sudden darting or freezing], and very elevated = 5 [hyperalert, excited, sudden bouts of running or body movements]). We assessed each animal’s reactivity to general stimuli ranging from no reaction to hyperactivity. Removal from the cage was assessed as: very easy = 0 (sits quietly, allows observer to pick it up), easy = 1 (vocalizations may occur, picked up without much resistance), moderately difficult = 2 (rears, often follows observer’s hand, vocalizations may occur), difficult = 3 (runs around cage, is hard to grab, with or without vocalizations), and very difficult = 4 (tail and throat rattles, with or without vocalizations, may attack hand). The handling reactivity was scored as: very low = 0 (totally limp or otherwise unresponsive), low = 1 (no resistance, easy to handle), moderately low = 2 (slight resistance, with or without vocalizations), moderately high = 3 (may freeze, be tense, or rigid in hand, with or without vocalizations), and high = 4 (squirming, twisting, or attempting to bite, with or without vocalizations).

### Sensorimotor domain

Sensorimotor responses to different kind of stimuli were used to detect severe sensory deficits. Pain perception was assessed by ranking the reaction to a tail pinch (no reaction = 0, slight reaction = 1 [animal displays little or no movement, turns, ambulates forward, or emits limited vocalization], moderate reaction = 2 [animals freezes, flinches, or vocalizes], exaggerated reaction = 3 [jumps, bites, or attacks, may also display frequent or constant vocalization]). Measuring of the response latency to a nociceptive (thermal stimulus) was performed by placing the animal on a heated (52 ± 1 °C) surface. The response to a mechanically produced “click” was quantified to assess audition and reactivity (no reaction = 0, slight reaction = 1 [some evidence that the noise was heard], freezes or flinches = 2 [actual muscle contractions], exaggerated reaction = 3 [jumps, bites, or attacks]).

### Autonomic domain and physiological domain

We assessed signs of autonomic reaction observing each animal for a minimum of 3 min in an open-field observation box. We quantified: the degree of lacrimation and salivation, with a range of severity scores from none to severe (none = 0, slight = 1, severe = 3); the presence or absence of piloerection and exophthalmos (not present = 0, present = 1); the number of urination and defecation episodes including polyuria and diarrhea; the pupillary function as indicated by constriction of the pupil in response to light (normal constriction of pupils = 0, partial or slow constriction of pupils = 1, lack of constriction of pupil = 3). Moreover, we also evaluated the incidence and severity of convulsions, tremors or degree of palpebral closure, ptosis, abnormal motor movements, both in the home cage and the open field (eyelids wide open = 0, eyelids slightly drooping = 1, eyelids drooping approximately halfway = 2, eyelids completely shut = 3). Bodyweight of all animals was measured within 3 days of arrival, prior to randomization, daily during repeat dosing phase, and daily from day 30 to end of study (withdrawal phase).

### Neuromuscular domain

In order to study the integrity of central nervous system motor function, we evaluated the incidence of gait abnormalities observed during the 3-min open-field observation period. The grading of any gait abnormality, with a range of severity scores from none to severe was performed as follows: no abnormality = 0; slightly impaired = 1 (i.e., any or all of the following may be evident: mild ataxia, rocks or lurches during ambulation, hunched or crouched body position, walks on tiptoe); moderately impaired = 2 (i.e., any or all of the following may be evident: marked ataxia, feet markedly point outward from the body, hindlimbs show exaggerated or overcompensated movements, drag, or are splayed); and severely impaired = 3 (i.e., any or all of the following may be evident: forelimbs drag or are unable to support weight, body drags or is flattened against surface). Forelimb and hindlimb grip strength were measured as previously described^36^. Quantitative measures of landing foot (hindfoot) splay were assessed. The descriptions and incidence of posture abnormalities were observed in the home cage as follows: no abnormality = 0; slightly impaired = 1 (i.e., any or all of the following may be evident: mild ataxia, rocks or lurches during ambulation, hunched or crouched body position, walks on tiptoe); moderately impaired = 2 (i.e., any or all of the following may be evident: marked ataxia, feet markedly point outward from the body, hindlimbs show exaggerated or overcompensated movements, drag, or are splayed); and severely impaired = 3 (i.e., any or all of the following may be evident: forelimbs drag or are unable to support weight, body drags or is flattened against surface). To assess locomotor activity, on the evening of day 30, animals were placed into the locomotor activity testing enclosure and monitored for 12 h to quantitatively measure the onset of acute signs of discontinuation syndrome. A 3-dimensional array of different activities was recorded for the most representative activity parameters which included: (i) basic movement calculated as number of horizontal ambulations (x + y plane) plus fine movement; this measure corresponds to all animal movements (general activity measure); (ii) fine movement calculated as number of times the animal moves without changing its whole body position, i.e., broke and cleared a single beam without ambulating; this measure corresponds to small animal movement such as grooming or head shaking; (iii) rearing event, assessed by quantifying the number of times rats break the vertical plane (z plane); corresponds to the number of rearing events; (iv) the cumulative distance (cm) traveled by the subject in a zone.

## Results

### Study 1

**Figure 3:**
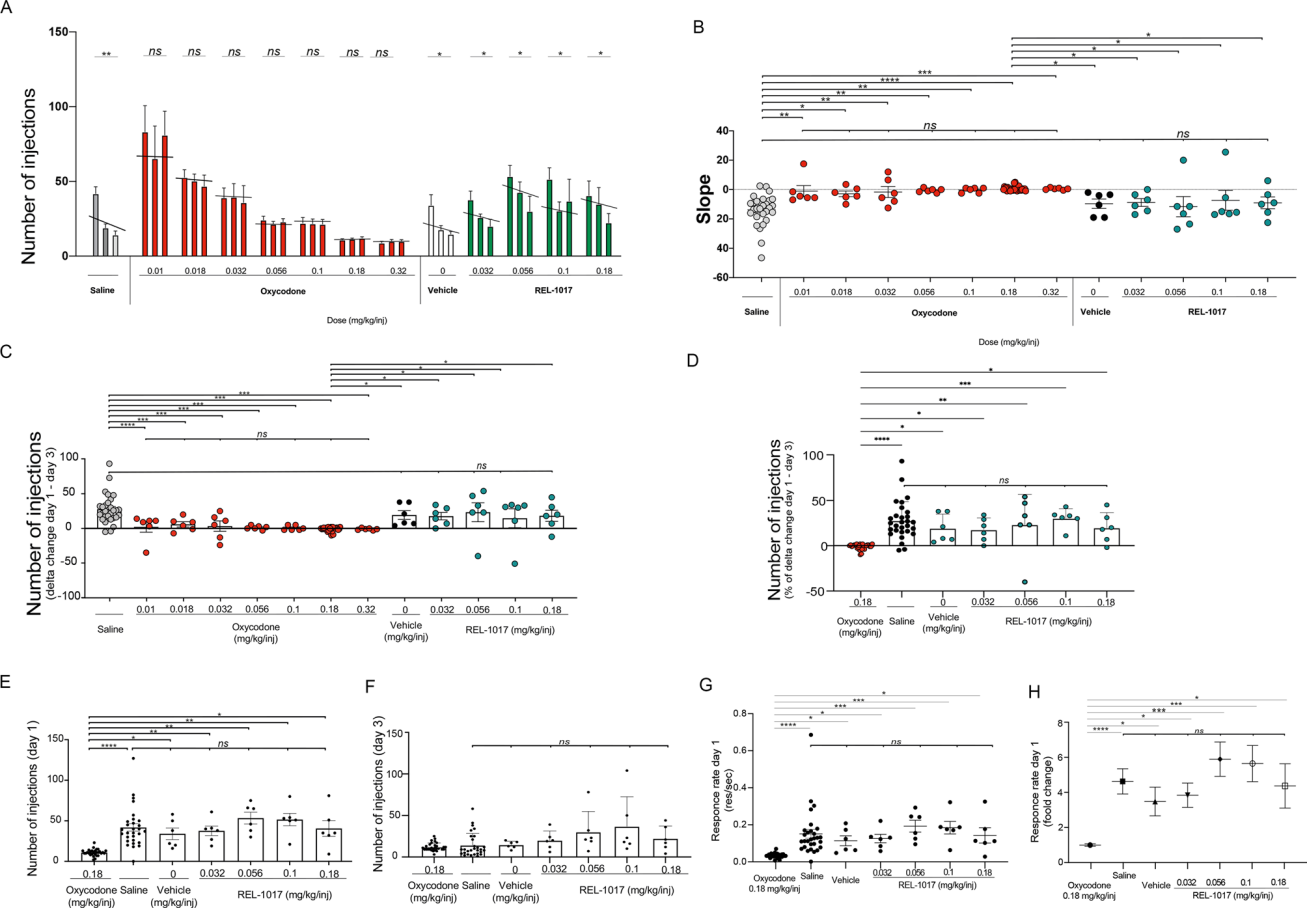
Self-administration of REL-1017 in rats previously trained to administer oxycodone in 3-day substitution study. (**A**) Drugs were self-administered via intravenous (IV) bolus injections utilizing the surgically implanted venous catheters connected to a Med-PC computer system. Injection requirement was FR10 (10 lever presses = 1 injection). Trained rats were tested with saline control (n = 29, gray bars) or with the 0.18 mg/kg/injection training/maintenance dose of oxycodone (n = 29). Following the establishment of oxycodone as a reinforcer, 6 rats per each treatment were tested with the positive control substance (0.01, 0.018, 0.032, 0.056, 0.10, and 0.32 mg/kg/injection oxycodone; red bars), and/or REL-1017 (0, 0.032, 0.056, 0.10, and 0.18 mg/kg/injection; green bars). Each group of 3 bars represents the results for Days 1, 2, and 3. Each bar represents the mean of ≥ 6 rats. All positive control groups exposed to any oxycodone dose maintained a stable number of infusions over 3 days (p = ns between injections at day 1 and 3 for all dosing groups). As expected for non-reinforcing stimuli in this experiment, saline, vehicle, and all REL-1017 doses showed a typical “extinction burst” pattern of responding, characterized by an initial rapid increase of lever-pressing on the first day followed by a downward staircase pattern of responses on the second and third day (*p < 0.05, **p < 0.01 between injections at day 1 and 3). (**B**) Comparison of the number of injections at day 1. (**C**) Comparison of the number of injections at day 3. (**D**) Delta changes between the injection number at day 1–3. (**E**) Percent of delta changes between the injection number at day 1–3. REL-1017 at all tested doses showed a pattern comparable to vehicle and saline. For each condition, the differences in the day-to-day pattern of injection during the test session and the linear regression functions (slopes) fitted to the total number of injections during each three-day interval calculated for each study item is shown in (**F**). The calculated slopes for saline (gray dots), vehicle (black dots), and REL-1017 at all doses (green dots) were not statistically different between each other. The calculated slopes for saline, vehicle, and REL-1017 at all doses were all statistically significantly different when compared to oxycodone (red dots; *p < 0.05; **p < 0.01; ***p < 0.001; ****p < 0.0001). All 4 doses of REL-1017 tested in this study demonstrated a downward staircase pattern resulting in negative sloped linear functions (saline = − 13.83; vehicle = − 9.667; RE-1017 0.032 mg/kg/injection = − 8.833; REL-1017 0.056 mg/kg/injection = − 11.67; REL-1017 0.1 mg/kg/injection = − 7.333; REL-1017 0.18 mg/kg/injection = − 9.083). All seven tested doses of oxycodone demonstrated a stable pattern resulting in neutral sloped functions (oxycodone 0.01 mg/kg/injection = − 1.083; oxycodone 0.018 mg/kg/injection = − 2.917; oxycodone 0.032 mg/kg/injection = − 1.667; oxycodone 0.056 mg/kg/injection = − 0.5833; oxycodone 0.1 mg/kg/injection = − 0.3333; oxycodone 0.18 mg/kg/injection = 0.569; oxycodone 0.32 mg/kg/injection = 0.5). (**G**) High rates of responding throughout the test sessions (responses/second) were observed in rats treated with saline (gray dots) and all doses of REL-1017 (green dots). The response rates for REL-21017 tested were significantly different from oxycodone (0.18 mg/kg/injection, red dots). (**H**) In particular, compared to the response rates for oxycodone 0.18 mg/kg/injection, saline had a 4.6-fold increase (p < 0.0001), REL-1017 vehicle had a 3.5-fold increase (p < 0.05), REL-1017 (0.032 mg/kg/injection) had a 3.8-fold increase (p < 0.001), REL-1017 (0.056 mg/kg/injection) had a 5.9-fold increase (p < 0.05), REL-1017 (0.1 mg/kg/injection) had a 5.6-fold increase (p < 0.05), and REL-1017 (0.18 mg/kg/injection) had a 4.4-fold increase (p < 0.05).

As shown in Fig. 3, rats exposed to all doses of oxycodone self-administered a number of injections, which was not statistically different between day 1 and day 3 [p = not significant (ns) comparing the number of injections at day 1 and day 3]. As expected for non-reinforcing stimuli in this assay, saline, vehicle, and all REL-1017 groups showed a typical “extinction burst” pattern of responding, which is characterized by an initial rapid increase of lever-pressing followed by decreased responding across sessions over 3 days. Extinction burst pattern of responding was assessed by comparing treatments based on: (i) the number of injections at day 1 versus day 3 (Fig. 3A); (ii) the calculated linear regression functions (slopes) fitting the number of injections during the 3-day interval for each treatment (Fig. 3B); (iii) the delta difference, and the delta percent change of the number of injections between day 1 and 3 (Fig. 3C-F); (iv) the response rates (number of self-injections/second) in the first minutes of the 1-h session of day 1 between the different treatments (Fig. 3G, H); (v) the total amount of drug administered and its relation with the number of injections (Fig. S2).

As shown in Fig. 3A, rats exposed to saline reached a peak response rate of 41.8 ± 24.6 (mean ± SD) injections at day 1, which progressively decreased to 19 ± 14 and 20 ± 24.6 at day 2 and 3 respectively (p < 0.005 between day 1 and 3). Similarly, REL-1017-treated groups achieved the highest injection rate in the first day. REL-1017 vehicle had a peak response rate of 34 ± 17.1 (mean ± SD) injections at day 1, which progressively decreased to 17.7 ± 6.6 and 14.7 ± 4.4 at day 2 and 3, respectively (p < 0.05 between day 1 and 3). REL-1017 (0.032 mg/kg/injection) reached a peak response rate of 37.7 ± 14.2 (mean ± SD) injections at day 1 decreasing to 25.8 ± 5.6 and 20 ± 11.3 at day 2 and 3, respectively (p < 0.05 between day 1 and 3). REL-1017 (0.056 mg/kg/injection) reached a peak response rate of 53.3 ± 17.9 (mean ± SD) injections at day 1 decreasing to 42.7 ± 17.1 and 30 ± 24.2 at day 2 and 3, respectively (p < 0.05 between day 1 and 3). REL-1017 (0.1 mg/kg/injection) reached a peak response rate of 51.5 ± 18.6 (mean ± SD) injections at day 1 decreasing to 30.3 ± 14.7 and 36.8 ± 14.6 at day 2 and 3, respectively (p < 0.05 between day 1 and 3). REL-1017 (0.18 mg/kg/injection) reached a peak response rate of 40.5 ± 24 (mean ± SD) injections at day 1 decreasing to 34.8 ± 24.7 and 22.3 ± 14.9 at day 2 and 3, respectively, (p < 0.05 between day 1 and 3).

In addition, to quantify differences in the day-to-day injection numbers during the test session, we also calculated the linear regression functions (slopes) fitting the number of injections during the 3-day interval (Fig. 3B, Table 3). Saline, vehicle, and REL-1017 at all doses demonstrated a pattern of progressive decrease in self-administration resulting in negative sloped linear functions (saline = − 13.83; vehicle = − 9.667; RE-1017 0.032 mg/kg/injection = − 8.833; REL-1017 0.056 mg/kg/injection = − 11.67; REL-1017 0.1 mg/kg/injection = − 7.333; REL-1017 0.18 mg/kg/injection = − 9.083). No significant differences between the calculated slopes for saline, vehicle, and REL-1017 at all doses were observed. On the contrary, all seven doses of oxycodone tested showed similar stable patterns of self-administration resulting in substantially neutral sloped functions (oxycodone 0.01 mg/kg/injection = − 1.083; oxycodone 0.018 mg/kg/injection = − 2.917; oxycodone 0.032 mg/kg/injection = − 1.667; oxycodone 0.056 mg/kg/injection = − 0.5833; oxycodone 0.1 mg/kg/injection = − 0.3333; oxycodone 0.18 mg/kg/injection = 0.569; oxycodone 0.32 mg/kg/injection = 0.5; p = ns). The calculated slopes for saline, vehicle, and REL-1017 at all doses were all statistically significantly different from oxycodone at 0.18 mg/kg/injection (Fig. 3B; p < 0.05). To better define the extent of responding in the 3-day substitution period, we also calculated the delta between day 1 and 3. The delta for any REL-1017 dose was not different from saline, while it was statistically different from oxycodone at all tested doses, both in absolute number as well as in % change (Fig. 3C, D). The total number of injections at day 1 and at day 3 was not different between saline and REL-1017 at any dose (Fig. 3E, F).

**Table 3:** Summary of the 3 day-to-day changes with corresponding slopes of regression lines.

Test condition	Dose mg/kg/injection	Slopes	Reinforcer (yes/no)	p (compare to saline)	p (compare to oxycodone 0.18)
Oxycodone	0.01	− 0.74	Yes	p < 0.0001	p = ns
	0.018	0.21	Yes	p < 0.001	p = ns
	0.032	− 1.39	Yes	p < 0.001	p = ns
	0.056	− 0.38	Yes	p < 0.001	p = ns
	0.1	0.126	Yes	p < 0.001	p = ns
	0.18	0.56	Yes	p < 0.001	
	0.32	0.52	Yes	p < 0.001	p = ns
Saline (Extinction)	0	− 18.83	No		p < 0.05
REL1017 Vehicle (Extinction)	0	− 9.67	No	p = ns	p < 0.05
REL1017	0.032	− 8.83	No	p = ns	p < 0.05
	0.056	− 11.67	No	p = ns	p < 0.05
	0.1	− 7.33	No	p = ns	p < 0.05
	0.18	− 9.01	No	p = ns	p < 0.05

To further assess the reinforcement properties of the tested drugs, we calculated the response rates (number of self-injections/second) in the first minutes of the 1-h session of day 1 (Fig. 3G). As expected for non-reinforcing stimuli, rats treated with saline and REL-1017 (0, 0.032, 0.056, 0.01, and 0.18 mg/kg/injection) engendered similar high response rates. The response rates of all tested drugs were significantly different from oxycodone (0.18 mg/kg/injection; Fig. 3G). In particular, the increases in the response rate as compared to oxycodone (0.18 mg/kg/injection) were 4.6 fold for saline (p < 0.0001), 3.5-fold for vehicle (p < 0.05), 3.8-fold for REL-1017 (0.032 mg/kg/injection) (p < 0.001), 5.9-fold for REL-1017 (0.056 mg/kg/injection) (p < 0.05), 5.6-fold for REL-1017 (0.1 mg/kg/injection) (p < 0.05), and 4.4-fold for REL-1017 (0.18 mg/kg/injection) (p < 0.05) (Fig. 3H).

Also, as shown in Fig. S2, the total amount of REL-1017 administered during the 3-day session increased progressively in the REL-1017 groups at increasing concentrations, without a significant change in the total number of injections (left panel, p = ns), as expected for non-reinforcing drugs. On the contrary, when oxycodone was self-administered, as expected for reinforcing opioid drugs, there was a progressive significant decrease in the total number of self-injections from the lowest (0.01 mg/kg/injection) to the highest (0.32 mg/kg/injection) oxycodone concentration (right panel, p < 001). These data indicate that REL-1017, at all tested doses, acted as a non-reinforcing stimulus.

### Study 2

**Figure 4:**
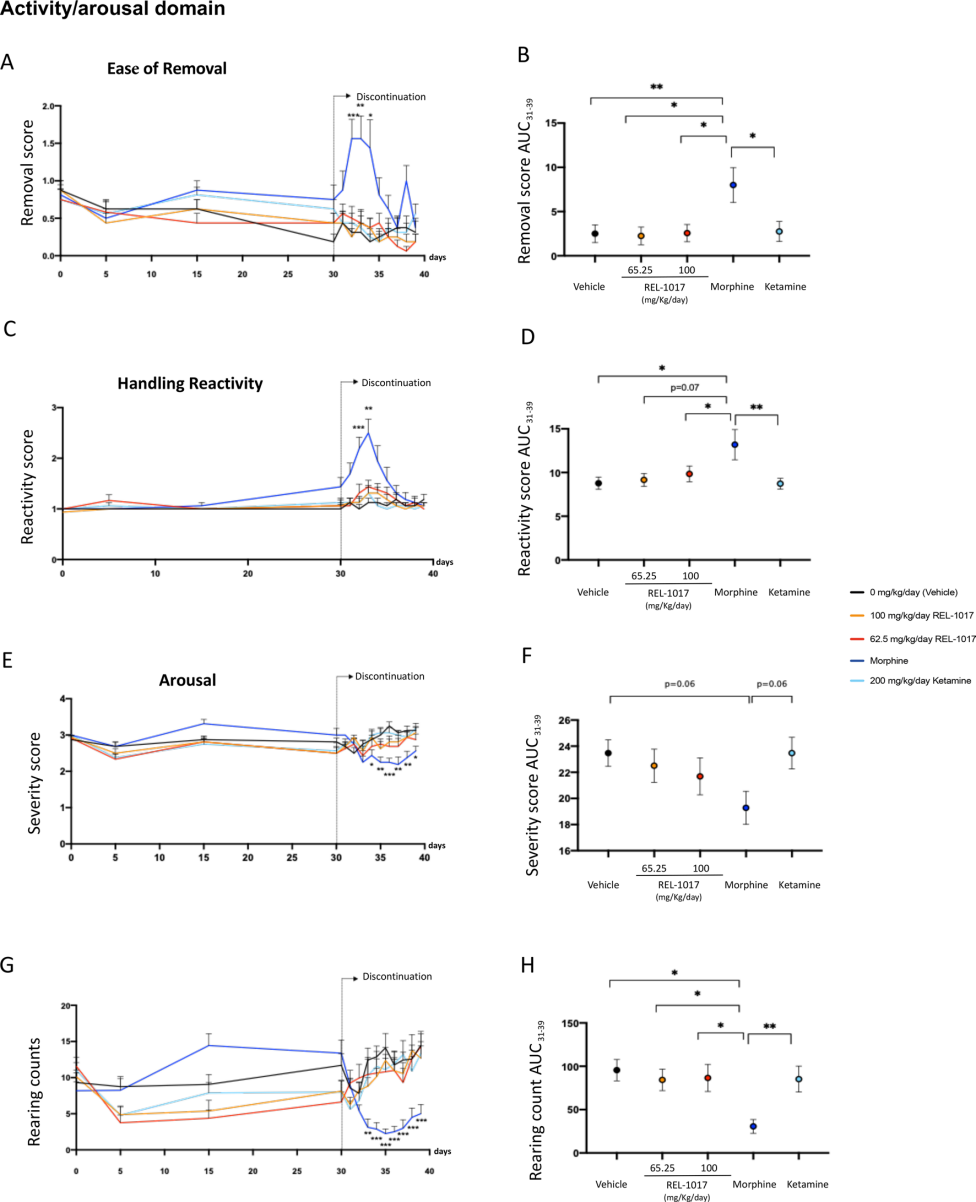
Discontinuation of chronic REL-1017 (esmethadone) administration did not cause modification in measures of excitability. Following cessation after 30 consecutive days of drug administration, measures of excitability, including ease of removal (**A**), handling reactivity (**C**), arousal (**E**), and rearing counts (**G**) were significantly different in morphine-treated rats (dark blue line) compared to vehicle (black line) and REL-1017-treated rats (yellow and red lines; *p < 0.05; **p < 0.01; ***p < 0.001; ****p < 0.0001). Area under the curve (AUC) values of ease of removal (**B**), handling reactivity (**D**), arousal (**F**), and rearing counts (**H**) were measured over the time interval 31–39 days. AUCs of morphine-treated rats (blue dot) were statistically different compared to AUCs of vehicle (black dot) and REL-1017- treated rats (yellow and red dots; *p < 0.05; **p < 0.01; ***p < 0.001; ****p < 0.0001).

**Figure 5:**
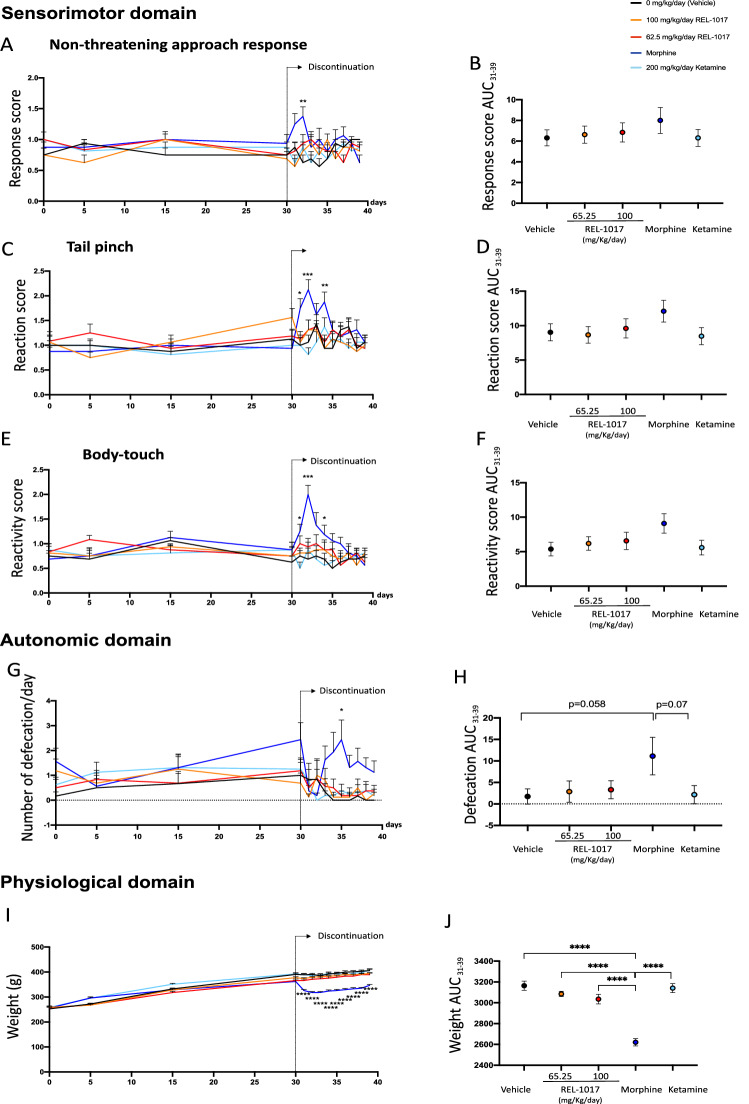
Discontinuation of chronic REL-1017 (esmethadone) administration did not cause modification in sensorimotor, autonomic and physiological measures. Following abrupt cessation after 30 consecutive days of drug administration, measures related to sensorimotor functions performed during the following 9 consecutive days, including non-threatening approach response (**A**), tail pinch (**C**), and simple body-touch (**E**) were significantly different in morphine-treated rats (dark blue line) compared to vehicle (black line) and REL-1017-treated rats (yellow and red lines *p < 0.05; **p < 0.01; ***p < 0.001; ****p < 0.0001). AUC values of non-threatening approach response (**B**), tail pinch (**D**), and simple body-touch (**F**) were measured over the time interval 31–39 days. No differences were observed between AUCs of morphine-treated rats (blue dot) to AUCs of vehicle (black dot) and REL-1017-treated rats (yellow and red dots). (**G**) Defecation (autonomic) and (**I**) body weight (physiological) measures obtained during the 9 days following abrupt cessation after 30 consecutive days of vehicle (black line) and REL-1017 (yellow and red lines) administration differed significantly from those obtained following abrupt cessation after 30 consecutive days of morphine (dark blue line; *p < 0.05 at day 5; ***p < 0.001, at day 1–9). The AUC value of defecation (**H**) measures over the time interval 31–39 days of morphine-treated rats (blue dot) showed a trend of increase compared to the AUC of vehicle (black dot, p = 0.058). The AUC value of body weight (**J**) measures over the time interval 31–39 days of morphine-treated rats (blue dot) were statistically decreased compared to the AUC of vehicle (black dot) and REL-1017-treated rats (yellow and red dots, ****p < 0.0001).

**Figure 6:**
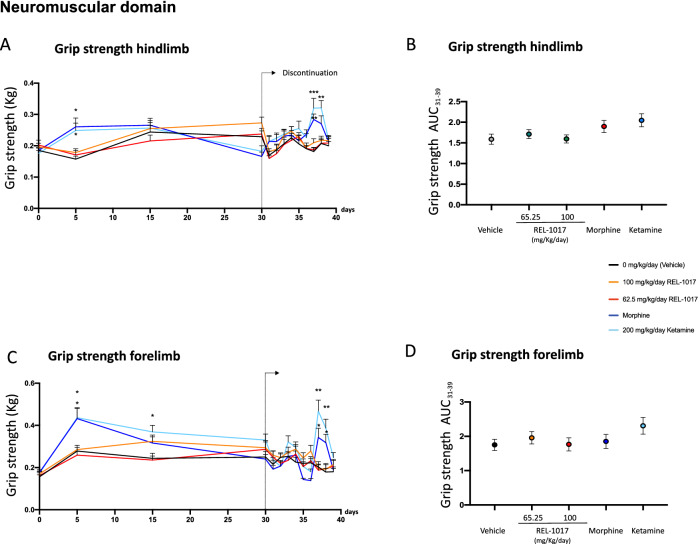
Discontinuation of chronic REL-1017 (esmethadone) administration did not cause modification in neuromuscular measures. Measurements of neuromuscular functions evaluated during the 9 days of discontinuation in rats treated for 30 consecutive days with morphine (dark blue line) demonstrated statistically significant changes in (**A**) hindlimb and (**C**) forelimb grip strength compared to vehicle (black line) and REL-1017 (yellow and red lines **p < 0.01; ***p < 0.001; at days 7 and 8). Ketamine-treated rats (light blue) showed significant changes in (**A**) hindlimb grip strength compared to vehicle and REL-1017 (*p < 0.05 at day 7). No differences were observed in the AUC values of hindlimb (**B**) and forelimb (**D**) grip strength measured over the time interval 31–39 days between morphine-treated rats (blue dot), vehicle (black dot) and REL-1017-treated rats (yellow and red dots).

The measures of the effects of drug discontinuation were based on behavioral assessment consisting of FOBs and measures of motor activity. No differences in mortality were observed in the six experimental groups (data not shown). In order to quantify the neurological effects of each drug exposure, we grouped the measured endpoints of each neurologic response and calculated specific scores. As previously reported, morphine-treated rats demonstrated statistically significant changes in clusters of withdrawal signs of the opiate-type over the 9 days of discontinuation (Figs. 4, 5, 6, Fig. S3). Also the area under the curve (AUC) values of ease of removal (Fig. 4B), handling reactivity (Fig. 4D), arousal (Fig. 4F), and rearing counts (Fig. 4H) were measured over the time interval 31–39 days. AUCs of morphine-treated rats (blue dot) were statistically different compared to AUCs of vehicle (black dot) and REL-1017-treated rats. The AUC value of defecation (Fig. 5H) measures over the time interval of 31–39 days for morphine-treated rats (blue dot) showed a trend of increase compared to the AUC of vehicle (black dot, p = 0.058). In addition to changes in excitability, sensorimotor, autonomic, and physiologic domains, morphine-treated rats also demonstrated statistically significant changes in measures of neuromuscular functions (Fig. 6A–D), including hindlimb grip strength (Fig. 6A; p < 0.01 at day 38 compared to control).

Upon abrupt discontinuation following 30 days of 200 mg/kg/day ketamine, rats exhibited a mild cluster of changes, as expected for this drug^37–39^. In particular, at days 37 and 38 there were changes in neuromuscular functions, measured as hindlimb and forelimb grip strength (p < 0.001 and p < 0.01, compared to control, respectively) (Fig. 6A,C). In contrast, upon abrupt discontinuation following 30 days of 62.5 and 100 mg/kg/day REL-1017, rats did not show signs ketamine- and morphine-like withdrawal syndrome.

In summary, none of the measures of opiate- or PCP-types of physical dependence and withdrawal were statistically different between groups treated with REL-1017 at 62.5 or 100 mg/kg doses and vehicle (p = ns). On the other hand, positive control groups showed statistically different scores compared to vehicle control and compared to 62.5 or 100 mg/kg REL-1017 in measures of excitability, sensorimotor, autonomic and physiologic domains, and neuromuscular functions. Overall, the data indicate that the performed testing batteries were adequate to identify opiate- or PCP-types of physical dependence and withdrawal. In this experimental setting, REL-1017 did not cause morphine-like or ketamine-like discontinuation syndromes following 30 days of drug exposure, indicating that in this study REL-1017 treatment does not cause withdrawal.

## Discussion

Racemic d,l-methadone, the 50/50 mixture of levomethadone and esmethadone, has been in clinical use for over 70 years for the treatment of pain and opioid use disorder. Levomethadone, the opioid active l-isomer of racemic methadone, is a full agonist of opioid receptors. According to a recent DEA statement “The d-isomer lacks significant respiratory depressant action and abuse liability…”^40^. Esmethadone binds to opioid receptors with a 20-fold lower affinity compared to levomethadone^41^ and does not appear to have meaningful opioid agonist effects in preclinical and clinical studies^3,4,8–14^. Furthermore, previous studies on methadone isomers suggest that esmethadone may have antagonistic actions for typical opioid agonist effects such as analgesia^13^, respiratory depression^42^, and subjective opioid effects^43^. Therefore, while levomethadone, the opioid active l-isomer of racemic methadone, is a full agonist at opioid receptors, esmethadone appears to be inactive and may even be antagonistic to levomethadone at opioid receptors. Both esmethadone and levomethadone bind to the MK-801-labeled uncompetitive site of NMDAR^5,44^. However, differently from MK-801, esmethadone does not cause Olney’s lesion in cortical neurons^45^. Esmethadone exerts in vitro NMDAR blocking effects comparable to other uncompetitive NMDAR antagonists in clinical use^46^. The hypothesized mechanism of action of esmethadone for MDD is via NMDAR antagonism and downstream mToRC1- and BDNF-dependent effects^2^.

Major depressive disorder (MDD) is the second leading cause of disability and chronic disease burden in the United States, among all medical conditions, as measured by “Disability-Adjusted Life Years”^47^. Available treatments for MDD include selective serotonin-reuptake inhibitors and atypical antipsychotics. Therapeutic effects with available treatments are generally delayed by several weeks and fail in approximately 50% of patients^48,49^. REL-1017 demonstrated robust, rapid, and sustained antidepressant effects in a recently completed Phase 2 clinical trial^3^ and is presently in Phase 3 clinical trials (ClinicalTrials.gov: NCT04688164; NCT04855747; NCT04855760).

Because of the gravity of the opioid crisis in the US, and the higher risk of substance abuse disorder in patients with MDD^50,51^, despite the lack of evidence for meaningful opioid agonist activity for esmethadone from previous preclinical and clinical studies^3,4,8–14^, we tested esmethadone in two established experimental models with predictive value for human abuse potential^52,53^. Since these experiments were performed in male rats, their relevance in female rats is unclear at the moment. We first explored reinforcing properties using the rat intravenous self-administration model. As previously described, following establishment of stable oxycodone self-administration (< 20% day-to-day variations, FR10 schedule), rats exposed to oxycodone over a 3-day session maintained a low, constant number of infusions (Fig. 3). In contrast, all groups treated either with saline, vehicle or REL-1017 at all dosages, showed a rapid increase in drug self-administration on day 1 followed by the typical “extinction burst” pattern of response on days 2 and 3^27,28^. In this experimental setting, the rapid increase followed by a very rapid decrease in drug self-administration observed in vehicle and REL-1017 groups, indicates that REL-1017 acts as a non-reinforcing stimulus. The rat self-administration model carries predictive value for drug abuse potential in humans^18,27,28^. In this study we have defined the lack of reinforcing properties of REL-1017 in terms of response patterns and compared those patterns of intake to a pharmacologically distinct reinforcing drug, such as oxycodone. Assessing the non-reinforcing properties of REL-1017 using different reinforcer drugs, including cocaine or phencyclidine, would be very informative. However, we decided to employ oxycodone because it is a well-known opioid agonist with high abuse liability. Previous studies evaluated NMDAR antagonists as reinforcer stimuli. Methadone is an opioid mu-receptor agonist that also binds with low affinity NMDA receptors as a non-competitive antagonist^5,54,55^. Both d- and l-isomers of methadone bind non-competitively to the MK-801-labeled site of the NMDAR with low micromolar IC50 values similar to that of ketamine and dextromethorphan, known NMDAR antagonists^5,7,56^ Rats self-administer (S)-ketamine but not (R)-ketamine. Antidepressant-like doses of (S)-ketamine, but not of (R)-ketamine, induce locomotor activity (in an opioid receptor-dependent manner), psychomotor sensitization, conditioned place preference, and selectively increases metabolic activity and dopamine tone in medial prefrontal cortex (mPFC) of rats^57^. The NMDAR receptor antagonists, dextromethorphan, and dextrorphan, demonstrated limited reinforcing efficacy in non-human primates trained to self-administer phencyclidine^58^ By contrast, phencyclidine, dexoxadrol, and dextrorphan maintained lever-press responding for drug deliveries in monkeys trained to self-administer racemic ketamine^59^. We then explored whether REL-1017 could cause withdrawal in a rat experimental model. Upon abrupt discontinuation, opioids cause a typical withdrawal syndrome; in contrast, NMDAR channel blockers produce a less well defined constellation of signs^59–65^. In this study, we assessed withdrawal by neurobehavioral tests consisting of several FOBs, including measures of activity/arousal, autonomic and physiological domains, and motor activity. Result showed that long-term (30 days) oral administration of REL-1017 at two different doses (62.5 or 100 mg/kg/day) did not produce signs of withdrawal following abrupt discontinuation. In contrast, the positive control drugs, morphine and ketamine, in this study, engendered withdrawal (Fig.4–6).

The neurobehavioral tests used for the withdrawal study were mostly based on opioid symptoms of withdrawal, with ketamine injection cessation only impacting hind and forelimb grip strengths, to a relatively small extent. Since d-methadone and ketamine share mechanisms of action, it remains to be ascertained if significant withdrawal would be observed for symptoms linked to cessation of treatment with psychoactive, non-competitive NMDA antagonists including depressive states, anhedonia, and disrupted social behavior. The present withdrawal study confirms an earlier similar study of morphine and esmethadone^10^ with the addition of data on lack of ketamine-like withdrawal.

## Conclusion

In summary, the results of these two studies confirm and extend previous data indicating a lack of reinforcing effect, physical dependence, and withdrawal of REL-1017 in animals^8–11^, in humans with addictive disorders^12–14^, in healthy human volunteers^4^, and in patients^3,66^. In these animal models designed to assess reinforcing and withdrawal potential of molecules with therapeutic potential, REL-1017 engendered neither a response pattern consistent with the known pharmacology and abuse of mu opiate agonists, like oxycodone, nor morphine-like or ketamine-like withdrawal after abrupt discontinuation. Overall, these experiments indicate that REL-1017 is not endowed with characteristics predictive of human abuse potential and support the development of REL-1017 for the treatment of MDD and potentially for the treatment of other diseases and disorders caused by pathological hyperactivation of NMDARs.

## Supplementary Information

Supplementary Legends.

Supplementary Figure S1.

Supplementary Figure S2.

Supplementary Figure S3.
