# Supplementary material for: REL-1017 (esmethadone; d-methadone) does not cause reinforcing effect, physical dependence and withdrawal signs in Sprague Dawley rats
Source: Sci Rep. 2022 Jul 6;12:11389. doi: 10.1038/s41598-022-15055-3 (PMC9259683; doi:10.1038/s41598-022-15055-3)
Supplement: Supplementary file 1 — Supplementary Legends. [file 41598_2022_15055_MOESM1_ESM.docx]

Supplementary File

REL-1017 (Esmethadone; d-Methadone) Does Not Cause Reinforcing Effect, Physical Dependence and Withdrawal Signs in Sprague Dawley Rats

Jack Henningfield,^1†^ David Gauvin,^2†^, Francesco Bifari,^3†^, Reginald Fant,^1^, Megan Shram,^4^ August Buchhalter,^1^ Judy Ashworth,^1^ Ryan Lanier,^1^ Marco Pappagallo,^5^ Charles Inturrisi,^5^ Franco Folli,^3^ Sergio Traversa,^5^ Paolo L Manfredi^5^

**Figure S1**: Normality test of data related to Study 1 and Study 2. Quantile-quantile (QQ) plots, showing the distribution of the data against the expected normal distribution. Normal (Gaussian) distribution was tested by using Shapiro-Wilk analysis. Results confirm a normal distribution of both the data related to Study 1 (A) and Study 2 (B).

**Figure S2:** REL-1017 (esmethadone) showed higher injection variability and response rate compared to oxycodone in rat self-administration study (A)

The extent of decreased responding (defined as number of injections) across the 3-day sessions was assessed by calculating the difference between the number of injections measured at day 1 and that at day 3. The bar graph showed that all doses of REL-1017 (green dots) were superimposable to saline (grey dots) and statistically different from oxycodone (red dots,

*=p<0.05; **=p<0.01; ***=p<0.001;****=p<0.0001). (B) Similar (p=ns) high rates of responding throughout the test sessions (responses/second) were observed in rats treated with saline (gray dots) and all doses of REL-1017 (green dots). The response rates for REL-21017 tested were significantly different from oxycodone (0.18 mg/kg/injection, red dots). (C) In particular, compared to the response rates for oxycodone 0.18 mg/kg/injection, saline had a 4.6- fold increase (p<0.0001), REL-1017 vehicle had a 3.5-fold increase (p<0.05), REL-1017 (0.032 mg/kg/injection) had a 3.8-fold increase (p<0.001), REL-1017 (0.056 mg/kg/injection) had a 5.9- fold increase (p<0.05), REL-1017 (0.1 mg/kg/injection) had a 5.6-fold increase (p<0.05), and REL-1017 (0.18 mg/kg/injection) had a 4.4-fold increase (p<0.05).

**Figure S3**: Increasing doses of REL-1017 (esmethadone) did not engender decreases in cumulative number of lever presses

Drug intake (black line, mean ± SEM) and cumulative number of lever presses (red line, mean ± SEM) is shown in the graph. The total number of injections remained constant between REL- 1017 groups at different doses and similar to that of vehicle and saline (left panel). In contrast, in the oxycodone groups, higher doses corresponded to a lower total number of injections (right panel).
