# Supplementary figures and images for: REL-1017 (esmethadone; d-methadone) does not cause reinforcing effect, physical dependence and withdrawal signs in Sprague Dawley rats
Source: Sci Rep. 2022 Jul 6;12:11389. doi: 10.1038/s41598-022-15055-3 (PMC9259683; doi:10.1038/s41598-022-15055-3)

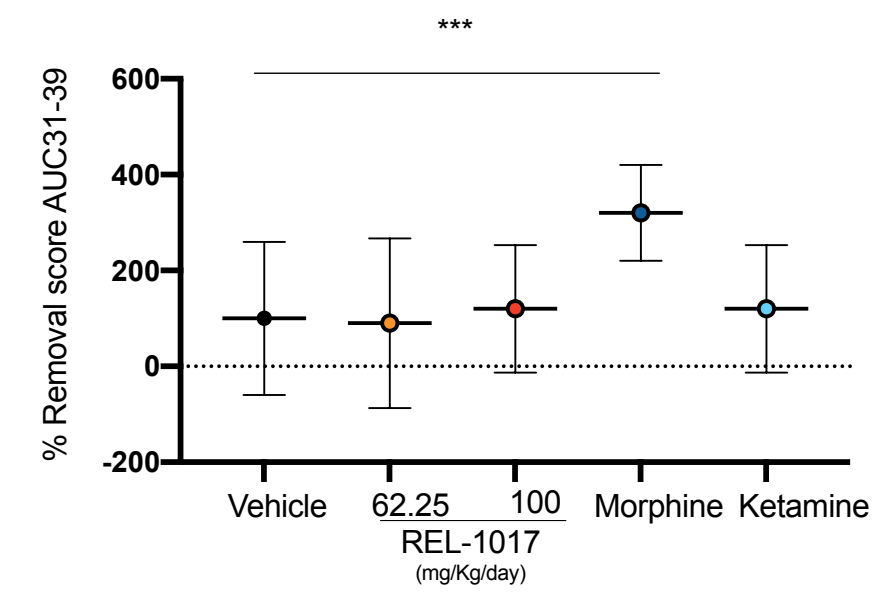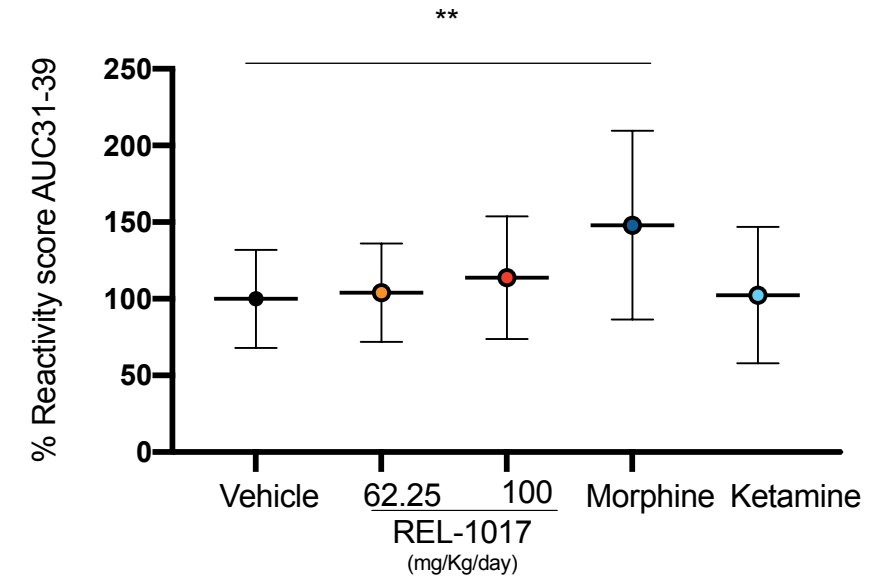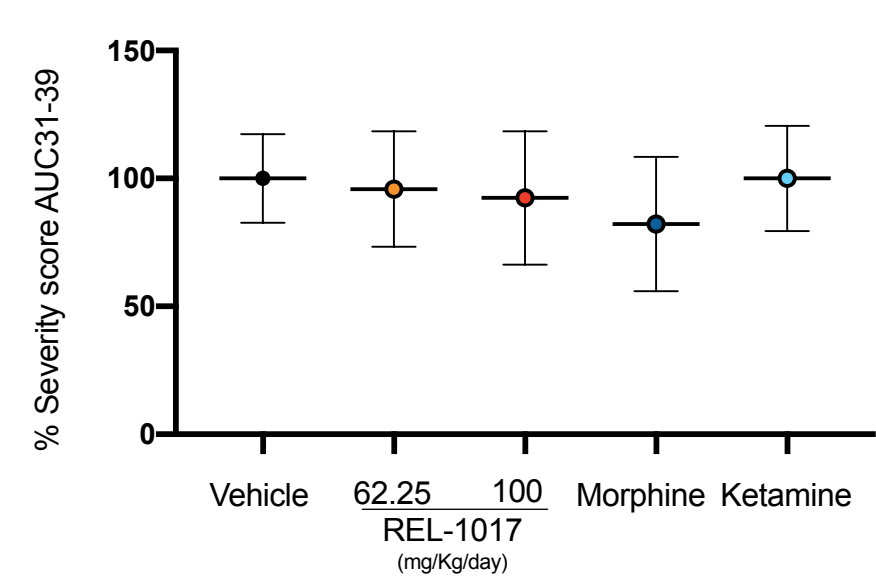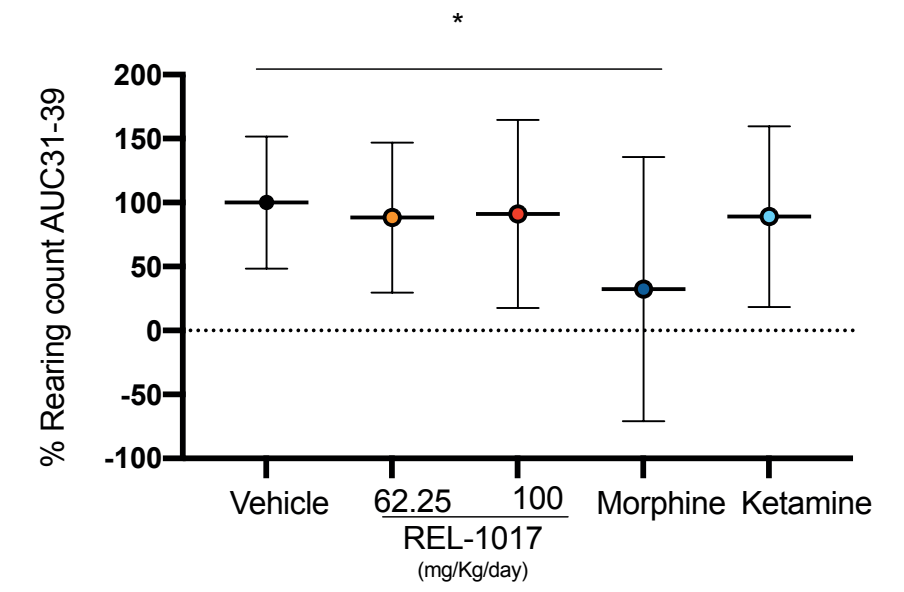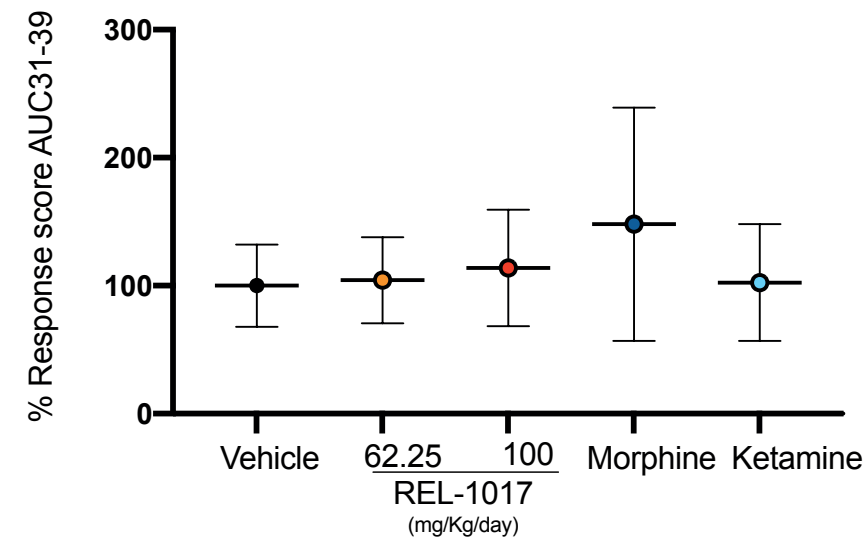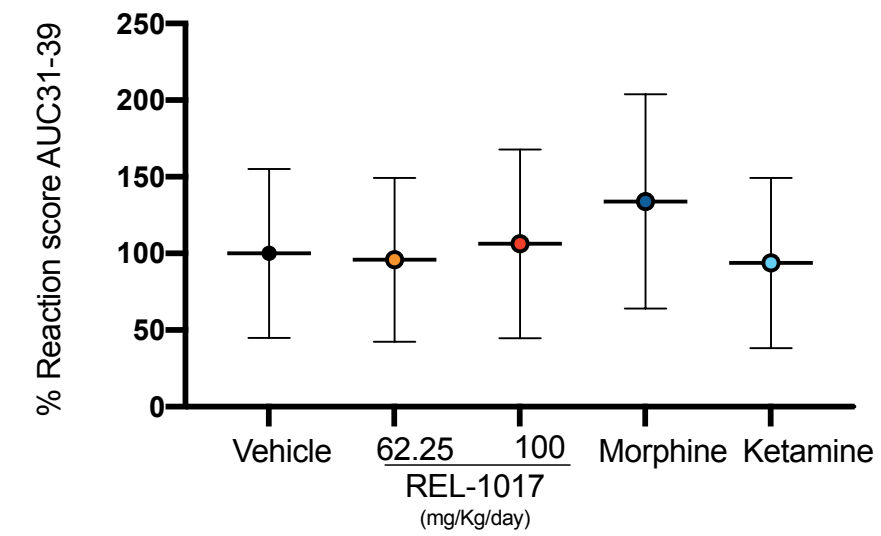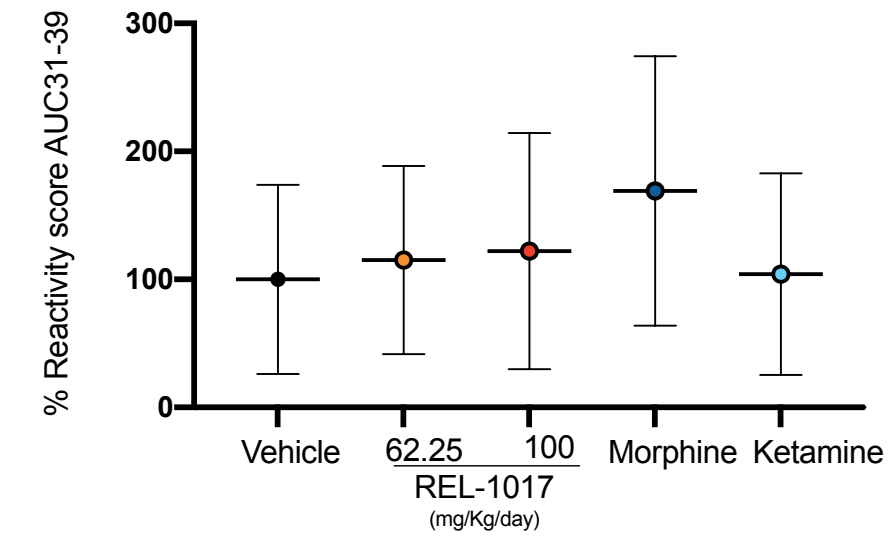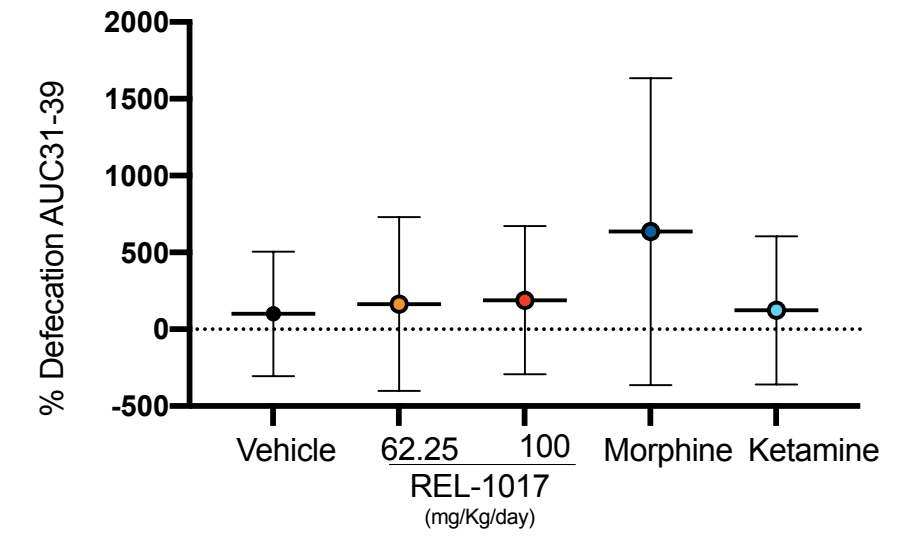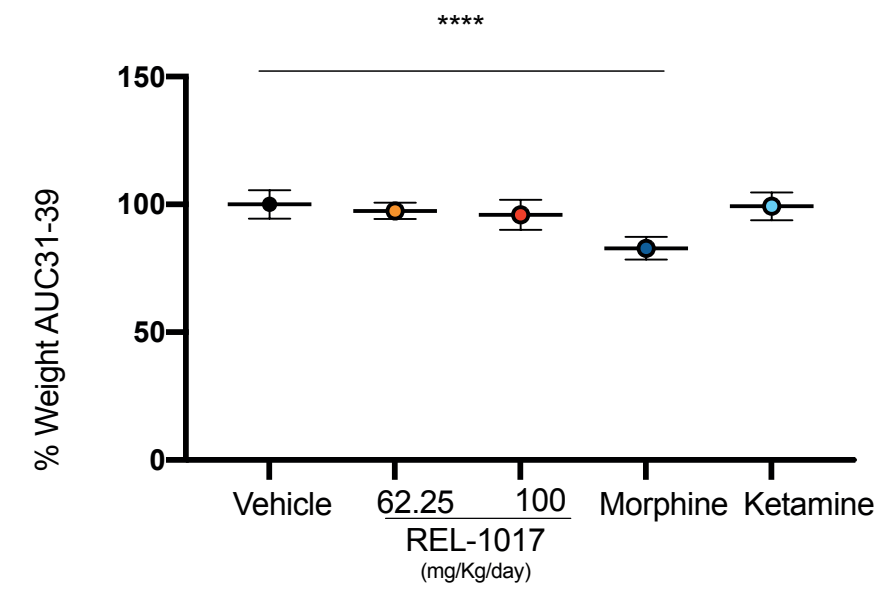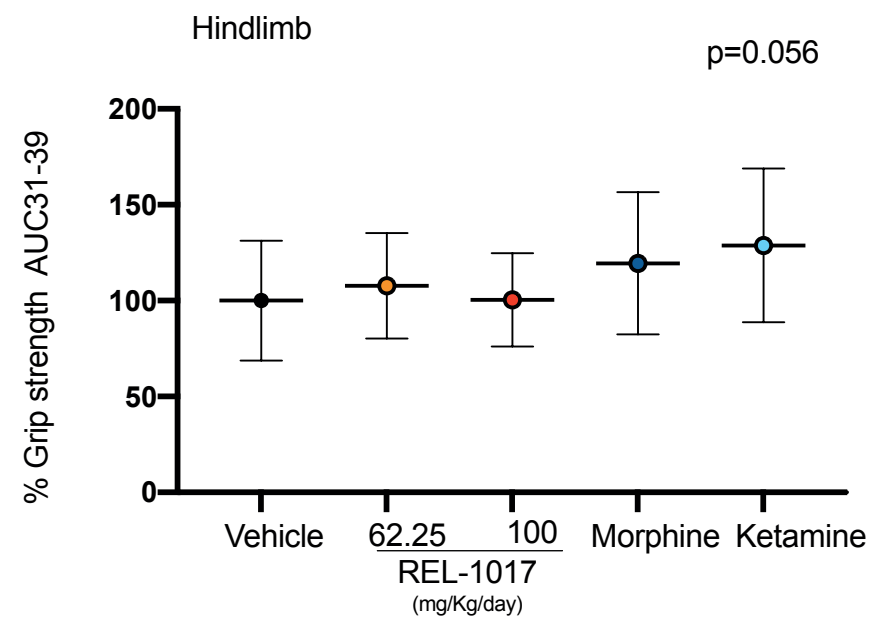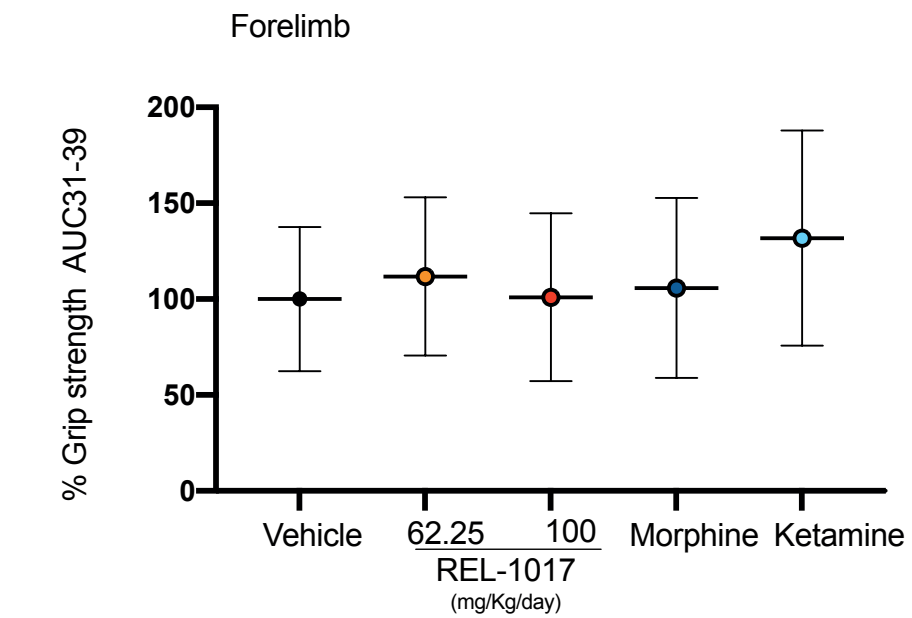

Supplement: Supplementary file 4 — Supplementary Figure S3. [file 41598_2022_15055_MOESM4_ESM.pdf]
